# Diet overlap among non‐native trout species and native cutthroat Trout (*Oncorhynchus clarkii)* in two U.S. ecoregions

**DOI:** 10.1002/ece3.7231

**Published:** 2021-02-07

**Authors:** Mario Minder, Emily R. Arsenault, Bolortsetseg Erdenee, Alain Maasri, Mark Pyron

**Affiliations:** ^1^ Department of Biology Ball State University Muncie IN USA; ^2^ Kansas Biological Survey and Department of Ecology and Evolutionary Biology University of Kansas Lawrence KS USA; ^3^ Department of Biodiversity, Earth & Environmental Science Drexel University Philadelphia PA USA; ^4^ The Academy of Natural Sciences of Drexel University Philadelphia PA USA; ^5^ Leibniz Institute of Freshwater Ecology and Inland Fisheries Berlin Germany

**Keywords:** cutthroat trout, diet, diet overlap, gut content analysis, native, non‐native

## Abstract

The invasion of freshwater ecosystems by non‐native species can constitute a significant threat to native species and ecosystem health. Non‐native trouts have long been stocked in areas where native trouts occur and have negatively impacted native trouts through predation, competition, and hybridization. This study encompassed two seasons of sampling efforts across two ecoregions of the western United States: The Great Basin in summer 2016 and the Yellowstone River Basin in summer 2017. We found significant dietary overlaps among native and non‐native trouts within the Great Basin and Yellowstone River Basin ecoregions. Three orders of invertebrates (Ephemeroptera, Trichoptera, and Diptera) composed the majority of stomach contents and were responsible for driving the observed patterns. Great Basin trout had higher body conditions (k), and non‐native Great Basin trout had higher gut fullness values than Yellowstone River Basin trout, indicating a possible limitation of food in the Yellowstone River Basin. Native fishes were the least abundant and had the lowest body condition in each ecoregion. These findings may indicate a negative impact on native trouts by non‐native trouts. We recommend additional monitoring of native and non‐native trout diets, regular invertebrate surveys to identify the availability of diet items, and reconsidering stocking efforts that can result in overlap of non‐native fishes with native cutthroat trout.

## Graphical abstract

Non‐native trout in the western United States pose a significant threat to native trout species. We found significant dietary overlaps among native and non‐native trouts within the Great Basin and Yellowstone River Basin ecoregions. Selectivity analysis also suggested that native trout were more selective and may be adapting to non‐native trout pressure by selecting different diet items.

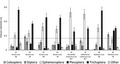

## 1 INTRODUCTION

The invasion of freshwater ecosystems by non‐native species presents a significant threat to native species and ecosystem health (Dick et al., 2017; Nico & Fuller, 1999; Pejchar & Mooney, 2009; Vilà et al., 2010). Salmonids have been introduced to freshwaters across the United States for over a century, mainly for increased angler opportunities and commercial fishing (Fuller et al., 1999; Halverson, 2010). These introductions often occur in locations where native salmonids are also present which can lead to a range of negative impacts on natives. In particular, brook trout (*Salvelinus fontinalis*) native to the Eastern United States and Canada, brown trout (*Salmo trutta*) native to Europe, Northern Africa, and western Asia, and rainbow trout (*Oncorhynchus mykiss*) native to Pacific ocean tributaries of North America and Asia are commonly stocked in streams and lakes by management agencies across the western United States (Fuller et al., 1999; Hutchings, 2014). Many of these waterbodies contain native cutthroat trout (*Oncorhynchus clarkii*) which are negatively affected by introductions of non‐native trouts (Fausch & White, 1981; Halverson, 2010; Peterson et al., 2004). The causes of these negative impacts vary from competition for habitat or food items, to predation and hybridization (McKelvey et al., 2016; Muhlfeld et al., 2014; Seiler & Keeley, 2009). Such impacts have been shown to cause reductions in juvenile cutthroat survival rates (Al‐Chokhachy & Sepulveda, 2019; Peterson et al., 2004), shifts in cutthroat movement patterns (McHugh & Budy, 2006), and potential resource competition as inferred by highly overlapping diets (Hilderbrand & Kershner, 2004; Tagliaferro et al., 2015).

Several species of non‐native trout in particular have been observed to affect the persistence of cutthroat trout. Peterson et al. (2004) found reductions of native cutthroat trout populations when in the presence of non‐native brook trout. Brown trout also negatively impact native cutthroat trout growth and are a potential predator of juveniles (Al‐Chokhachy & Sepulveda, 2019; McHugh & Budy, 2006; McHugh et al., 2008). Further, the introduction of rainbow trout is a unique conservation threat due to rainbow trout hybridization with cutthroat trout that produces viable offspring (Allen et al., 2016; Allendorf et al., 2001; Hitt et al., 2003). These hybrid offspring tend to have reduced fitness, yet reproduce naturally, increasing the likelihood of further hybridization of native cutthroat trout.

Dietary analysis is a common method of quantifying dietary overlap, predatory interactions, and possible competition among native and non‐native fish species (Griffith, 1974; Hilderbrand & Kershner, 2004; Sampson et al., 2009; Tab or & Wurtsbaugh, 1991). Metrics, like Schoener's similarity index or nonmetric multidimensional scaling (NMDS), allow identification of dietary overlap among species and identify potential competition that can be predictive of impacts between native and non‐native species (Declerck et al., 2002; Pilger et al., 2010; Sampson et al., 2009; West et al., 2003). Selectivity indices comparing dietary item abundances compared with environmental abundances are useful indicators of potential competition and changes in diets due to a variety of factors (season, elevation, presence of other fish species, etc.) (Buxton et al., 2017; Falke et al., 2020; Lowe et al., 2000; Mischke et al., 2003).

Native Bonneville cutthroat trout (*Oncorhynchus clarki utah*), a subspecies of cutthroat trout, occur in the Great Basin and have adapted to the fluctuating extreme hydrology of streams that are characteristic of this system (Behnke, 1992). The Great Basin of the western United States covers parts of seven states (Arizona, California, Idaho, Nevada, Oregon, Utah, and Wyoming) and is the largest endorheic watershed in North America (Sigler & Sigler, 1987). Native Yellowstone cutthroat trout (*Oncorhynchus clarki bouvieri*) are the most abundant and wide‐ranging subspecies of cutthroat trout and occur throughout the watershed (Varley & Gresswell, 1988). The Yellowstone River Basin is located across North Dakota, Wyoming, and Montana, spanning ~34,000 square miles (WGFD, 2017). This ecoregion is categorized as a forest steppe ecoregion due to its high elevation and grassland plains. While research has been done on both subspecies of cutthroat trout separately (Al‐Chokhachy & Sepulveda, 2019; Gresswell, 2011; Hilderbrand & Kershner, 2004; McHugh et al., 2008), little work has been done to directly compare the diets of these subspecies when in the presence of non‐native trout species.

This study encompassed two month‐long sampling efforts across two US ecoregions: the semiarid steppe (Great Basin) and the forest steppe (Yellowstone River Basin). Our aim was to quantify dietary overlap and dietary variability among native and non‐native trout species occurring in both ecoregions. We hypothesized that (a) within ecoregions, native cutthroat trout would have specialist diets with high levels of dietary selectivity and lower levels of dietary overlap with non‐native trouts, (b) non‐native trout would have more generalist diets with lower levels of selectivity and higher levels of dietary overlap, and (c) cutthroat trout would maintain low variability in dietary selectivity between ecoregions, while non‐native trout species would have higher variability in dietary selectivity between ecoregions.

## 2 METHODS

### 2.1 Study area

**Figure 1:**
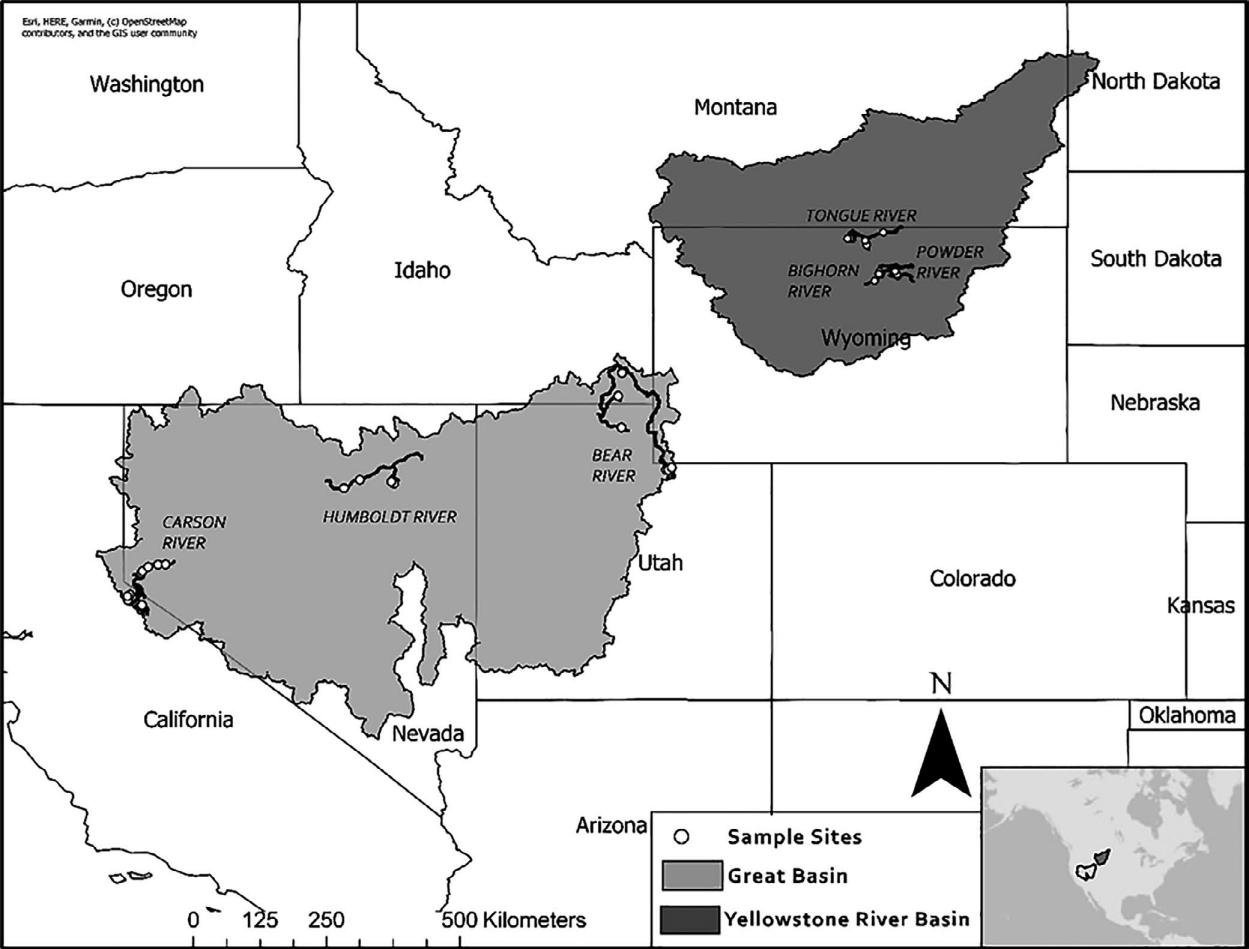
Sampled sites in the Bear, Carson, and Humboldt Rivers in the US Great Basin (light gray; *n* = 23) and in the Bighorn, Powder, and Tongue Rivers in the Yellowstone River Basin in Wyoming (dark gray; *n* = 20)

We sampled 23 sites in three rivers of the Great Basin: the Bear, Carson, and Humboldt Rivers, and 20 sites in three rivers of the Yellowstone River Basin: the Bighorn, Powder, and Tongue Rivers (Figure 1). Sites were selected as part of a larger macrosystems project (MACRO macrorivers.ku.edu) using the GIS‐based tool RESonate to characterizes river segments using valley‐scale hydrogeomorphic variables (Williams et al., 2013). Sites were chosen to maximize variability in hydrogeomorphology and ensure that sites accurately represent the broad geographic ecoregions that we sampled. Details for Great Basin site characteristics and designations can be found in Maasri et al. (2019).

### 2.2 Diet collections

Fish collections were performed during July and August 2016 in the Great Basin and July 2017 in the Yellowstone River Basin. At each site, fishes were collected from reaches measuring 20 times the average wetted width of the stream (Patton et al., 2000). Fishes were collected with one‐pass backpack electrofishing supplemented with hook and line and seining, following American Fisheries Society standard collection protocols and local states collecting regulations (Bonar et al., 2009). All fishes collected were identified to species, weighed (g), and measured for standard length (mm). When available, ten fish from each species at each site were randomly selected and sacrificed for gut content analysis (Ball State University IACUC #126193). Stomachs were removed for preservation in 10% formalin. For all fish, only the stomach was examined to minimize bias caused by digestibility of diet items (Sutela & Huusko, 2000). A quantitative survey (abundance per m^2^) of benthic invertebrates that was conducted at all study sites at the time of fish collection (Unpublished dataset, B. Erdenee 2016) was used to determine the proportional environmental abundance of each diet item for selectivity analyses.

### 2.3 Diet analysis

Gut content analysis was based on methods used in Minder et al. (2020). Guts (esophagus to pyloric valve) were evacuated of all contents and weighed, and contents were examined under a dissecting scope. All invertebrates were identified to family using Merritt and Cummins (1996). We grouped invertebrates to order, and orders that represented <1% of the total number of diet items were grouped into a single “other” category. To reduce bias caused by moisture trapped in samples, contents of each gut were dried at 50° for 48 hr and weighed after identification to determine gut fullness (FI) (Parker, 1963). FI was only calculated for fish that had nonempty guts.

FI was measured using the dry weight of the stomach content approach:

$$FI\;=\frac{\mathrm{FWd}}{\mathrm{Ww}}x\;100.$$

Where *FI* is the percentage of total weight contributed by the gut contents, FWd is the dry weight of the gut contents, and Ww is the wet weight of the fish (Schleuter, 2007).

Body condition of fish was calculated using Fulton's Condition Factor (K) (Nash et al., 2006).

$$K=100\left(\frac{W}{L^{3}}\right).$$

Where *K* is Fulton's condition factor, *L* is the length of the fish in centimeters, and *W* is the wet weight in grams. For salmonids, *K* > 1.4 is considered good condition and *K* < 1 is considered poor condition (Barnham & Baxter, 2003).

Calculations of frequency of occurrence (*FO*) and mean prey abundance (*Ni*) were used to quantify diets of individual fishes. *FO* was calculated as follows:

$$FO=\left[\frac{F_{i}}{P}\right]\times \;100.$$

where *FO* is the occurrence of a prey item $F_{i}$ divided by the number of nonempty guts (*P)*. The metric *FO* describes the percentage of individuals that have consumed a specific food item. While this metric does not provide details on amounts of items consumed, it is robust to limitations of other diet analysis challenges such as differences in prey condition and presence of unidentifiable tissues. (Baker et al., 2014; Buckland et al., 2017).

Mean prey abundance (*N_i_*) was used to compare feeding behavior and diet composition among fishes (Macdonald & Green, 1983). *N_i_* was calculated as follows:

$$N_{i}=\frac{1}{P}\times \left(\Sigma \left[\frac{N_{\mathrm{ij}}}{\Sigma N_{\mathrm{ij}}}\right]\right).$$

Where *N_i_* is the mean number of prey *i* consumed, *N_ij_* is the number of prey *i* in a single predator *j*, and *ΣN_ij_* is the sum of all the prey in a single predator gut *j*.

Dietary behavior was quantified with the Chesson's α electivity index (Chesson, 1978):

$$\alpha =\frac{\left(r_{i}/p_{i}\right)}{\sum \left(r_{i}/p_{i}\right)}$$

where *r_i_* is the proportion of the diet item consumed by an individual fish, *p_i_* is the proportional environmental abundance of the diet item at the capture site, and *n* is the number of prey item categories. If α = 1/*n,* the item in the diet is equal to its proportion in the environment, and we can assume that the item has been randomly selected. If α > 1/*n*, then the diet item has been positively selected for, and if α < 1/*n*, then that diet item has been avoided. Environmental abundances for diet items were calculated for each sample site and then averaged for each fish species to ensure that site‐specific selectivity was maintained.

Finally, we calculated the degree of diet overlap to assess diet similarities among fish species at a site using numerical gut content abundances. Mean proportional abundances were compared among species using Schoener's similarity index:

$$C=1‐\frac{1}{2}\left(\Sigma \left|P_{x,i}‐P_{y,i}\right|\right).$$

Where C is the Schoener's similarity index metric, and *P_x_*_,_ *_i_* and *P_y,i_* are the proportions of diet item *i* in the gut of species *x* and *y*, respectively (Schoener, 1970). This index ranges from 0 to 1 with values of 0 indicating no diet overlap and values of 1 indicating a complete overlap of diet items. Schoener's index values higher than 0.6 or lower than 0.4 are generally considered ecologically relevant (Childs et al., 1998; Muth & Snyder, 1995; Wallace, 1981).

### 2.4 Statistical analysis

We used one‐way ANOVA and Tukey's post hoc tests to compare mean variation for gut fullness among fishes. Statistics were calculated using R version 3.4.3 (R Core Team, 2017). We used nonmetric multidimensional scaling (NMDS) with Bray–Curtis distance to examine relationships among fish diet contents by species. NMDS generates an ordination based on a specified number of dimensions and attempts to meet the conditions of a rank similarity matrix (Clarke, 1993). NMDS also produces stress values to quantify the effectiveness of an ordination for pattern analysis, with values below 0.2 considered to be compliant (Clarke, 1993). This method uses ranked distances and is therefore useful for data that fail to meet the assumptions of normality (Clarke & Warwick, 2001; McCune et al., 2002). Pearson's correlations were conducted using NMDS scores from fish diets and the abundance of invertebrate orders, and these coefficients were plotted to show the degree of association between fish species and diet items (West et al., 2003).

We used ANOSIM to test the null hypothesis that there is no difference in fish gut contents among the assemblage of species (Clarke, 1993). To ensure diets from fish collected by angling did not differ from other methods, we also tested for differences within species by each sampling method. ANOSIM produces a test statistic (R) that quantifies the observed differences between test variables. R is expressed as a number between 1 and −1, which can be interpreted as maximum dissimilarity between groups and maximum similarity between groups, respectively (Clarke, 1993). An R value of 1 indicates complete dissimilarity between two groups, an R value of 0 is interpreted as complete similarity among groups, and a negative R value suggests that there is more similarity between groups than within groups (Clarke, 1993).

## 3 RESULTS

We captured and measured 1,102 fishes and processed 464 guts from four species of trout in the U.S. Great Basin and Yellowstone River Basin (Table 1). Tiger trout (*Salmo trutta × Salvelinus fontinalis*) were found in both ecoregions but excluded for low presence (3/23 sites (*n* = 22) in the Great Basin and 1/20 sites (*n* = 2) in the Yellowstone River Basin) and Paiute sculpin (*Cottus beldingii*) (*n* = 60) were captured in the Great Basin, but were excluded from these analyses due to a complete absence in the Yellowstone River Basin. We analyzed two native subspecies of cutthroat trout: Bonneville cutthroat trout (*Oncohynchus clarkii utah*) and Yellowstone cutthroat trout (*Oncorhynchus clarkii bouvieri*) and three non‐native species: brook trout (*Salvelinus fontinalis*), brown trout (*Salmo trutta*)*,* and rainbow trout (*Oncorhynchus mykiss*). Of the 464 fishes analyzed, 15 individuals (~3%) had empty stomachs with all but one collected from the Great Basin. Despite the increased occurrence of empty guts, Great Basin trout had significantly higher body condition factors than trout in the Yellowstone River Basin (ANOVA; *F*_7_ = 211, *p* < .001, Table 2). We did not see any significant differences in diets within species based on capture method (ANOSIM; *p* > .05).

**Table 1:** Total number of trout sampled, number of sites they were present in, and number of guts analyzed by ecoregion

Species	Great Basin			Yellowstone River Basin		
	Sampled *n*	Sites present	Guts analyzed	Sampled *n*	Sites present	Guts analyzed
Brook trout	201	12	95 (10)	206	18	93 (1)
Brown trout	84	10	42 (2)	244	9	82 (0)
Bonneville cutthroat*	43	4	26 (1)	–	–	–
Yellowstone cutthroat*	–	–	–	108	7	34 (0)
Rainbow trout	71	10	48 (1)	61	12	44 (0)

The number of empty guts in fish analyzed is in parentheses. Asterisks (*) represent native species.

**Table 2:** Biological data for trout used for diet analysis in the Great Basin (GB) and Yellowstone River Basin (YB)

Species (Ecoregion)	*n*	Standard Length ± (SE) (mm)	Mass (g)	Fulton's (K)
Brook trout (GB)	95	138.3 ± (3.7)	53.9 ± (4.3)	1.65 ± (0.03)
Brook trout (YB)	93	167.6 ± (4.2)	58.8 ± (3.8)	1.07 ± (0.01)
Brown trout (GB)	42	120.2 ± (6.9)	40.7 ± (8.9)	1.62 ± (0.02)
Brown trout (YB)	82	194 ± (6.2)	86.6 ± (6.2)	0.99 ± (0.01)
Bonneville cutthroat trout (GB)*	26	173 ± (11.5)	105.5 ± (15.9)	1.54 ± (0.04)
Yellowstone cutthroat trout (YB)*	34	167.1 ± (11.5)	72.5 ± (13)	0.98 ± (0.02)
Rainbow trout (GB)	48	198.2 ± (10.2)	175.5 ± (25.1)	1.64 ± (0.03)
Rainbow trout (YB)	44	183.4 ± (5)	69.4 ± (4.6)	1.06 ± (0.02)

Salmonids with *K* > 1 are considered to be in poor condition and *K* > 1.4 are in good condition (Barnham & Baxter, 2003). Variation represents mean ± SE. Asterisks (*) represent native species.

### 3.1 Gut fullness

**Figure A1:**
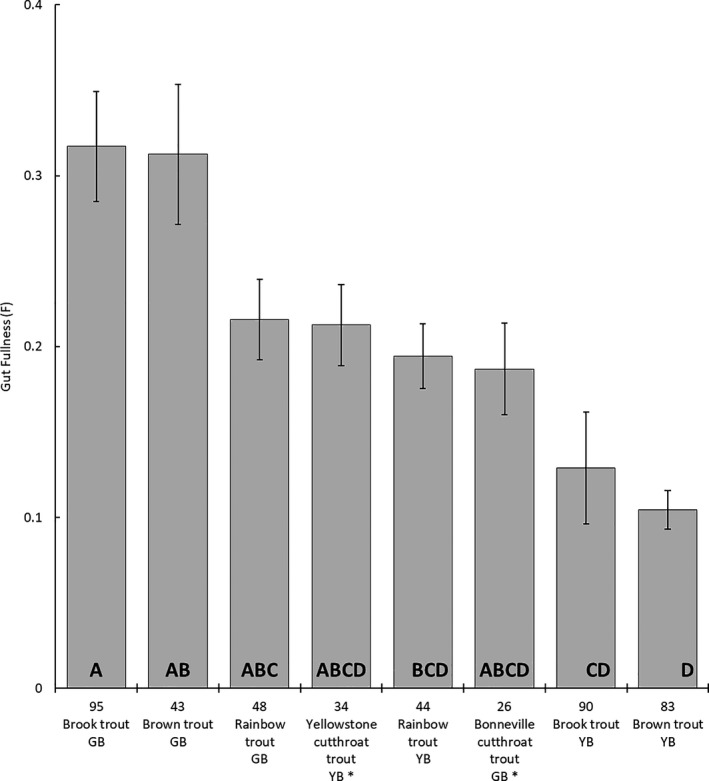
Mean (± SE) gut fullness index (FI) with post hoc Tukey pairwise comparisons for fishes in the Great Basin (GB) and Yellowstone River Basin (YB). FI was calculated using dry weight of contents and wet weight of fish. Fish with empty guts were excluded from the analysis. Species that share a letter are not significantly different from Tukey's multiple comparisons. Sample sizes are above species labels. Asterisks (*) represent native species

Gut fullness was significantly higher in Great Basin fishes (ANOVA *F*_1_ = 50.5, *p* < .001) than in the Yellowstone River Basin (see Figure A1 in Appendix). A Tukey post hoc analysis showed distinct patterns among species: Great Basin brook trout had the highest gut fullness, and Yellowstone River Basin brown trout had the lowest gut fullness (*p* < .001). Great Basin brook trout and brown trout had significantly higher gut fullness than the same species in the Yellowstone River Basin, while rainbow trout and both subspecies of cutthroat trout did not differ among ecoregions. In the Great Basin, non‐native brook trout and brown trout had higher gut fullness values than native cutthroat but in the Yellowstone River Basin this relationship was reversed.

### 3.2 Diet contents

**Figure 2:**
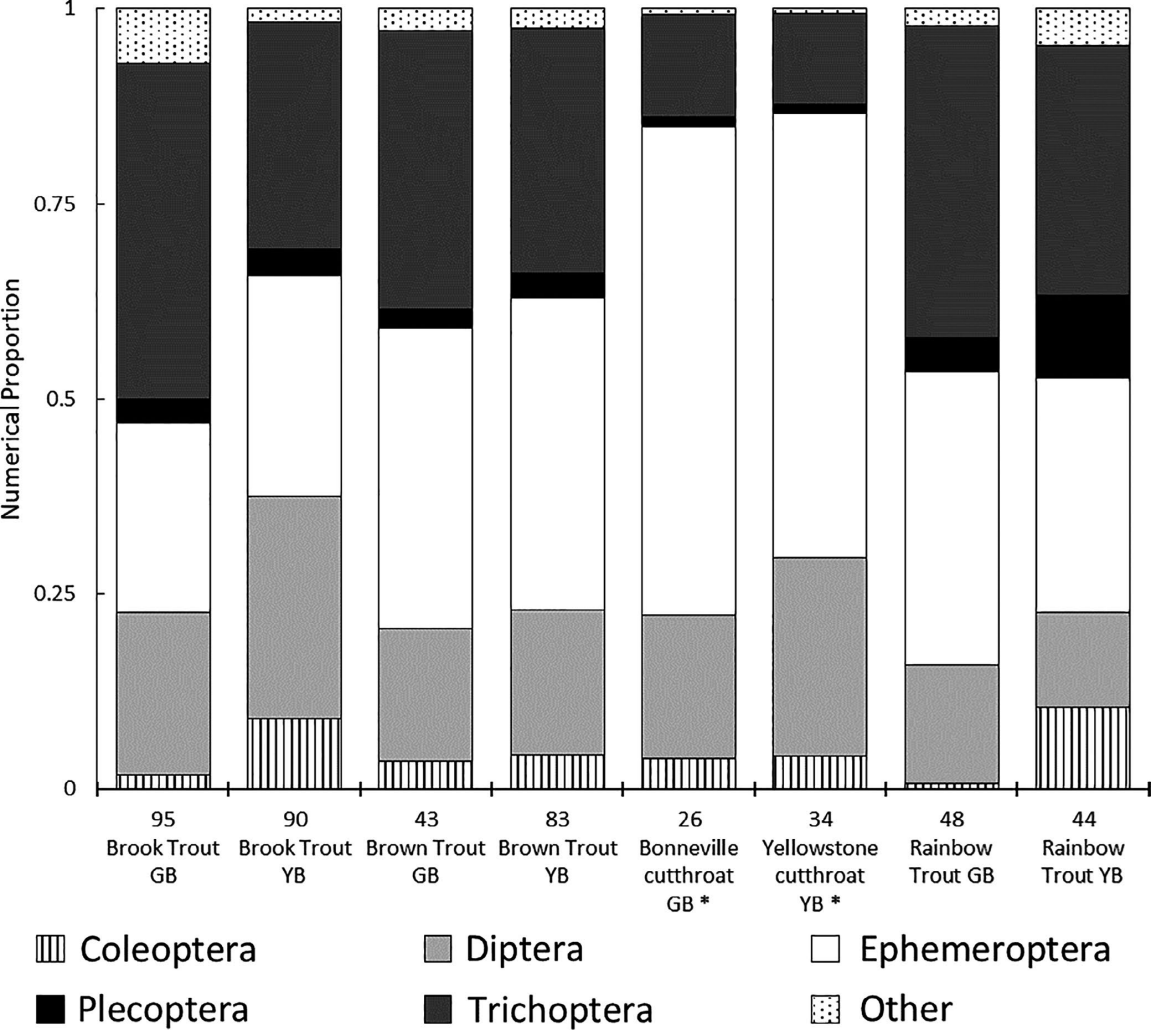
Diet composition by proportion for trout sampled. A total number of food items were converted to proportions to compare diets among species. Sample sizes are above species labels. Debris was not included due to its nondiscrete nature. Ecoregions are Great Basin (GB) and Yellowstone River Basin (YB). Asterisks (*) represent native species

**Figure B1:**
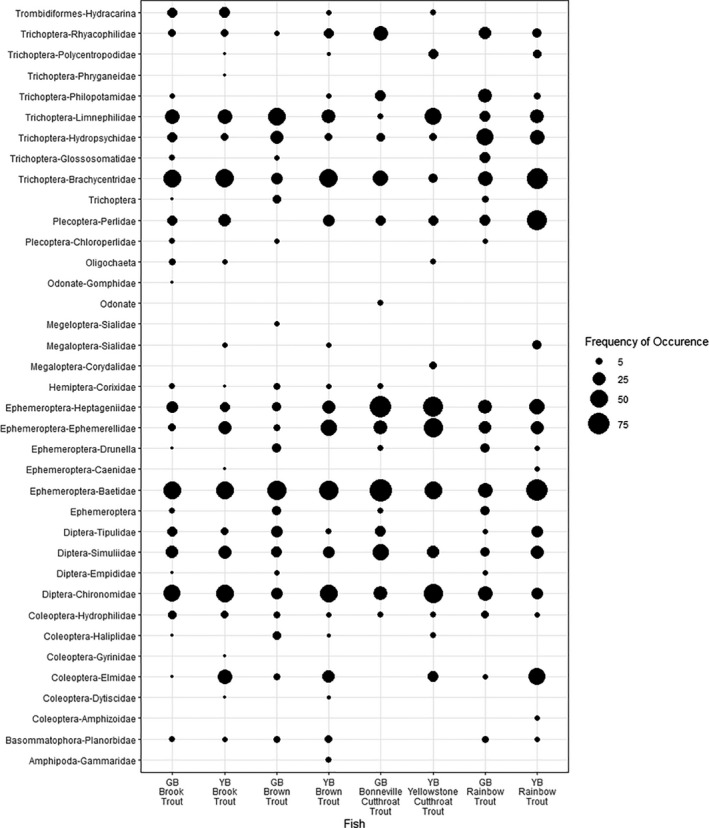
Bubble plot indicating the frequency of occurrence (FO) of invertebrates in the diets of fish species from our sampling areas. The orders represent diet items that were not able to be identified to family based on condition in the gut. Larger bubbles represent higher frequencies and lack of bubbles represent a lack of that item in the diet of a fish species

**Figure C1:**
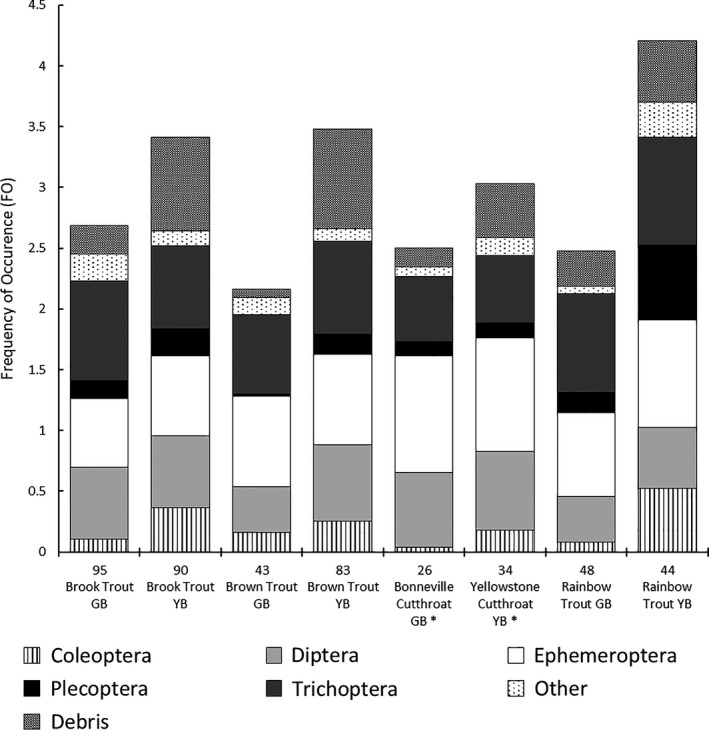
Frequency of occurrence (FO) of major diet items by fish species. Bar height represents frequency of diet items. Sample sizes are above species labels. Ecoregion abbreviations are Great Basin (GB) and Yellowstone River Basin (YB). Asterisks (*) represent native species

The frequency of occurrence (FO) of diet items provided the simplest quantification of diets. When we categorized diet contents to family level, we detected a wide range of relationships among fish species (see Figure B1 in Appendix). Several insect families were shared among all fish species, and many families occurred in diets in low abundances or were only present in a few fishes. When we grouped families into orders, comparisons of diets among fish species were apparent (see Figure C1 in Appendix). Debris was present in all diet samples but varied considerably by species and ecoregion. Yellowstone River Basin trout had higher mean FO scores than Great Basin trout, suggesting a greater variety of diet items. When contents were quantified numerically, debris was not included due to its nondiscrete properties (Figure 2). Cutthroat trout had greater proportions of Ephemeroptera and lower proportions of Trichoptera than non‐native trouts. Brook trout and rainbow trout had the highest variation among ecoregions.

**Figure 3:**
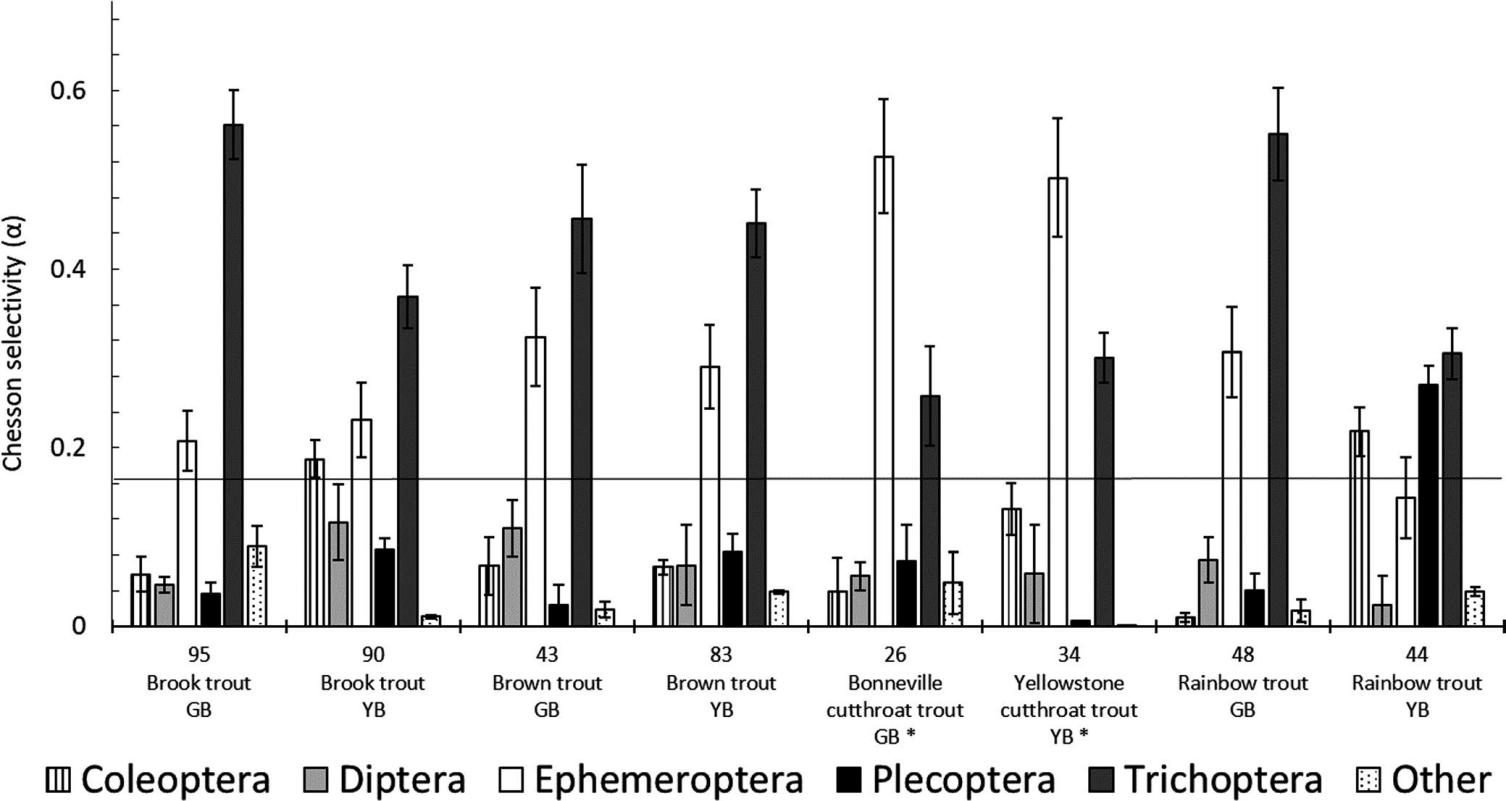
Diet composition by proportion for trout sampled. A total number of food items were converted to proportions to compare diets among species. Sample sizes are above species labels. Debris was not included due to its nondiscrete nature. Ecoregions are Great Basin (GB) and Yellowstone River Basin (YB). Asterisks (*) represent native species

The trends in mean prey abundances we observed were supported by a Chesson's electivity analysis (Figure 3). Both subspecies of cutthroat selected Ephemeroptera in higher frequencies than Trichoptera. Rainbow trout and brook trout selectivity varied between ecoregions, whereas cutthroat and brown trout selectivity did not differ between ecoregions.

### 3.3 Diet overlap

We quantified diet overlap among fish species using Schoener's similarity index and confirmed our interpretations using NMDS and ANOSIM. Only two species pairs did not overlap significantly (Table 3). Four species comparisons resulted in scores > 0.9, with the highest overlap being observed between brown trout and brook trout in the Great Basin (0.95). Cutthroat trout had the lowest total overlap scores, and brown trout had the highest.

**Table 3:** Schoener's similarity matrix (C) for all species combinations in the Great Basin (GB) and Yellowstone River Basin (YB)

Species	Brook (GB)	Brook (YB)	Brown (GB)	Brown (YB)	Bonneville cutthroat* (GB)	Yellowstone cutthroat* (YB)	Rainbow (GB)
Brook (YB)	0.81						
Brown (GB)	0.84	0.82					
Brown (YB)	0.82	0.85	0.95				
Bonneville cutthroat (GB)*	**0.59**	0.66	0.74	0.77			
Yellowstone cutthroat (YB)*	0.60	0.71	0.73	0.76	0.93		
Rainbow (GB)	0.85	0.78	0.94	0.90	0.69	0.67	
Rainbow (YB)	0.78	0.84	0.83	0.84	0.61	**0.59**	0.81

Note

Scores are the result of comparisons of mean numerical proportions of gut contents for each diet item. Scores > 0.6 and < 0.4 are considered ecologically important and represent high and low levels of diet overlap. Scores < 0.6 are in bold, scores > 0.9 are underlined, pairs that co‐occurred spatially are in shaded boxes. Asterisks (*) represent native species.

**Figure 4:**
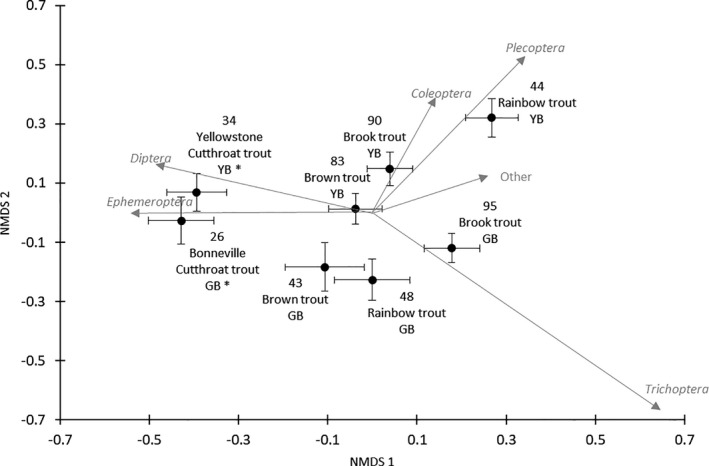
Biplot of NMDS ordination for the first and second axis correlation vectors represents diet items for insect orders collected during summer 2016 in the US Great Basin and summer 2017 in the Yellowstone River Basin. Points and bars are the mean and standard error for each taxon collected. The stress for this analysis was 0.13. Ecoregions are Great Basin (GB) and Yellowstone River Basin (YB). Sample sizes are above species labels. Asterisks (*) represent native species

Mean prey abundances were used as inputs for NMDS. The NMDS analysis converged in 3 dimensions with a stress of 0.13, meeting the threshold for useable pattern analysis (Figure 4). Low standard error values in NMDS represent low variability in diet (specialists), and large standard error represents high diet variability (generalists). Fishes that overlap in Figure 4 had high dietary overlap. The first and second dimension resulted in distinct patterns among species, specifically the separation of non‐native trout by ecoregion on the second axis (Figure 4).

ANOSIM results suggested similar diet overlap trends as the NMDS plots. In the Great Basin and the Yellowstone River Basin ecoregions, brook trout, brown trout, and rainbow trout diets did not differ significantly (ANOSIM; Great Basin, *p* = .149; Yellowstone River Basin, *p* = .334). In the Great Basin, Bonneville cutthroat trout diets significantly differed from brook trout diets (ANOSIM; R = 0.152, *p* = .002). Within the Yellowstone River Basin, the greatest dissimilarity in diets was between Yellowstone cutthroat trout and rainbow trout (ANOSIM; R = 0.187, *p* < .001). We did not find any significant differences when we compared Yellowstone cutthroat trout that co‐occurred with non‐native species to Yellowstone cutthroat trout that were collected at sites that did not have non‐native trout present (ANOSIM; R = 0.108, *p* = .028).

When we compared species across ecoregions, we could quantify relationships by geographical location. Cutthroat trout and brown trout diets did not differ significantly across ecoregions (ANOSIM; cutthroat trout, *p* = .617; brown trout, *p* = .038). Brook trout and rainbow trout diets differed significantly, but these differences were minor and likely ecologically insignificant (ANOSIM; R = 0.043, *p* < .001; R = 0.045, *p* = .004) (Anderson & Walsh, 2013; Clarke, 1993).

Pearson's correlations (represented as vectors) for invertebrate order abundances in fish guts with NMDS axis scores displayed a number of dietary trends (Figure 4). Cutthroat trout diets were significantly correlated with Diptera and Ephemeroptera abundances on all three NMDS axes. Only rainbow trout diets were correlated with Coleoptera abundances on the third NMDS axis. Additional correlation results were not significant. Rainbow trout and brook trout in the Great Basin were significantly correlated with Trichoptera abundance. However, in the Yellowstone River Basin, rainbow trout and brook trout were significantly correlated with Coleoptera and Plecoptera abundances. Brown trout diets in both basins were not significantly correlated with any invertebrate group abundances.

## 4 DISCUSSION

We found significant dietary overlaps among native and non‐native trouts within the Great Basin and Yellowstone River Basin ecoregions. Three orders of invertebrates (Ephemeroptera, Trichoptera, and Diptera) composed the majority of stomach contents and were responsible for driving the observed patterns. Great Basin trout had higher body conditions, and non‐native Great Basin trout had higher gut fullness values than Yellowstone River Basin trout overall. Low body condition and gut fullness may indicate food limitation in the Yellowstone River Basin, and when considered in combination with dietary overlap, can suggest evidence for interspecific competition (Angermeier, 1982; Hasegawa, 2016). The differences in body condition may be partially related to the elevation of the sites. Fishes at higher elevations tend to have lower body condition than those at lower elevations (Shuai et al., 2018), and the average elevation for our Yellowstone basin sites (2,518 m) was higher than the average of our Great Basin sites (2,149 m).

Native cutthroat trouts had the lowest dietary overlap values in each ecoregion and were less selective than non‐native species, therefore, partially supporting our first and second hypotheses. Native cutthroat trout and non‐native brown trout diets were not significantly different among ecoregions partially supporting our third hypothesis. Brook trout and rainbow trout diets differed by ecoregion, also supporting our third hypothesis, but observed variation was minor and can be expected to be ecologically irrelevant (Anderson & Walsh, 2013; Clarke, 1993).

Native cutthroat trout were the least abundant trout species in the Great Basin. They occurred at the fewest number of sites in both ecoregions and predominately co‐occurred with a non‐native species of trout. This co‐occurrence may result in competition because brook trout, brown trout, and rainbow trout have been shown to negatively impact native cutthroat trout through competition for habitat and food (Peterson et al., 2004; Seiler & Keeley, 2009). Cutthroat trout selectivity for Ephemeroptera over Trichoptera separated their diets from all non‐native trout, which selected primarily Trichoptera over Ephemeroptera. The observed differences between cutthroat trout diets and selectivity and non‐native trout diets and selectivity may be the result of cutthroat trout avoiding competition (McHugh & Budy, 2006) .

To avoid competition, cutthroat trout can also segregate from brown trout by elevation when they co‐occur, with cutthroat occupying higher elevations (Ernesto & Budy, 2005). The only sites where non‐native trout were absent occurred in the Yellowstone River Basin. The cutthroat trout at these sites appeared to select for different diet items, but their diets were not significantly different from cutthroat trout that co‐occurred with non‐native trout. Due to our small sample size of Yellowstone cutthroat trout (14 co‐occurring and 20 isolated individuals), targeted research in this region that focuses on the difference in the diets of cutthroat in the presence and isolated from non‐natives would be beneficial in determining specific behavioral and physical changes that may be occurring due to invasions.

Brown trout were the only species that did not co‐occur with other trout species in at least one sample site, in both ecoregions. Yellowstone River Basin brown trout had poor body condition (*k* ≤ 1) and the lowest gut fullness of all trout. Great Basin brown trout were less abundant but had excellent body condition (*k* ≥ 1.6) and the second highest overall gut fullness values of all sampled fishes. These physical factors suggest that Yellowstone River Basin brown trout may be experiencing intraspecific competition that affects growth (Johnsson et al., 1999; Olsén et al., 1996). Despite these differences in body condition and abundance, brown trout maintained similar diets and had almost identical selectivity values in both ecoregions. This consistency may be evidence of the specific ecological niche of brown trouts and given brown trouts ability to successfully establish in invaded areas may be predictive of establishment in our sample areas (Hasegawa, 2020; Lobón‐Cerviá & Sanz, 2017; Valiente et al., 2010).

Brook trout occurred in the greatest number of sample sites in both ecoregions. They were the only trout species at multiple Great Basin sites, but always co‐occurred with other trout species in the Yellowstone River. Brook trout displayed lower levels of selectivity in the Yellowstone River Basin than in the Great Basin. Interactions between cutthroat trout and brook trout are well‐studied, with resulting negative impacts on native fishes through competition and predation (Dunham et al., 2002; Hilderbrand & Kershner, 2004; Peterson et al., 2004). We did not detect predation upon cutthroat trout; however, predation may only occur seasonally when juvenile cutthroat trout are present and vulnerable (Gregory & Levings, 1996; Stapp & Hayward, 2002). Brook trout occurring in both ecoregions only had moderate diet overlaps, suggesting that direct dietary competition is unlikely.

Between ecoregions, the diets and selectivity of Rainbow Trout was the most variable of all trout species and Rainbow trout only displayed moderate diet similarity with cutthroat trout, limiting, therefore, the likelihood of dietary competition. However, the ability of rainbow trout to hybridize and negatively impact native cutthroat populations should be considered when assessing their potential impact on native fishes. While we did not identify any hybrid trout in our samples, maintaining isolated populations of native cutthroat trout and removing rainbow trout in areas where these species overlap is critical to prevent hybridization and loss of native populations (Muhlfeld et al., 2009).

The Great Basin and Yellowstone River Basin ecoregions contained co‐occurring native and non‐native trouts. Native fish were the least abundant and had the lowest body condition in both ecoregions. Non‐native trout had higher dietary overlap with other non‐native trout taxa than with native cutthroat trout, and native cutthroat trout selected different diet items than non‐native trout, suggesting low likelihood of dietary competition between native and non‐native fishes. The consistency in cutthroat trout diets among ecoregions and their differences from non‐native trout diets could be a result of adapting diet to avoid competition (McHugh & Budy, 2006). However, non‐native trout may still impact native trout through hybridization, predation, and habitat exclusion (Fausch & White, 1981; Hitt et al., 2003; McHugh et al., 2008). While we did not see significant differences between native cutthroat trout that were isolated and those that co‐occurred, future studies that expand the sample size and target isolated native cutthroat trout may clarify this trend. We hope that future isotope analysis will allow us to clarify and support the conclusions we made based on our gut content analysis. We recommend additional monitoring of diets among native and non‐native trout, regular invertebrate surveys to identify the availability of diet items, as well as reconsidering stocking efforts that can result in overlap of non‐native fishes with native cutthroat trout.

## CONFLICT OF INTEREST

We have no conflicts of interest to declare for this paper.

## AUTHOR CONTRIBUTIONS

**Mario Minder:** Conceptualization (equal); Data curation (lead); Formal analysis (lead); Investigation (equal); Writing‐original draft (lead); Writing‐review & editing (equal). **Emily R. Arsenault:** Conceptualization (equal); Investigation (equal); Writing‐original draft (equal); Writing‐review & editing (equal). **Bolortsetesg Erdenee:** Investigation (equal); Methodology (supporting); Writing‐review & editing (equal). **Alain Maasri:** Funding acquisition (lead); Investigation (equal); Writing‐review & editing (equal). **Mark Pyron:** Conceptualization (equal); Funding acquisition (lead); Investigation (equal); Methodology (equal); Project administration (equal); Writing‐original draft (equal); Writing‐review & editing (equal).

## Data Availability

Data to support this paper are stored on Dryad (https://doi.org/10.5061/dryad.8931zcrpz).
